# Hydroxytyrosyl Oleate: Improved Extraction Procedure from Olive Oil and By-Products, and In Vitro Antioxidant and Skin Regenerative Properties

**DOI:** 10.3390/antiox8070233

**Published:** 2019-07-20

**Authors:** Cinzia Benincasa, Chiara La Torre, Pierluigi Plastina, Alessia Fazio, Enzo Perri, Maria Cristina Caroleo, Luca Gallelli, Roberto Cannataro, Erika Cione

**Affiliations:** 1 CREA–Research Centre for Olive, Citrus and Tree Fruit, C.da Li Rocchi, 87036 Rende (CS), Italy; 2 Department of Pharmacy, Health and Nutrition Sciences, University of Calabria, Via Pietro Bucci, 87036 Arcavacata di Rende (CS), Italy; 3 Clinical Pharmacology and Pharmacovigilance Operative Unit, Department of Health Science, University of Magna Graecia, Mater Domini Hospital Catanzaro, 88100 Catanzaro, Italy

**Keywords:** hydroxytyrosol, keratinocytes, microRNA, olive mill waste water, phenolics, pomace, reactive oxygen species, superoxide dismutase

## Abstract

Recently, we identified hydroxytyrosyl oleate (HtyOle) in the by-products of olive oil, pomace and olive mill waste water (OMWW). Herein, we report that HtyOle is more accurately quantified by extracting the phenolic fraction from both matrices by using aqueous methanol (80%). By applying this method, HtyOle was also detected in extra virgin olive oil (EVOO). Since olive oil is used in the preparation of many cosmetic formulations, we explored the antioxidant capacity of HtyOle in human keratinocytes. Formation of reactive oxygen species (ROS) and malondialdehyde (MDA), as well as activity of Glutathione-S-transferase (GST) and superoxide dismutase (SOD) were decreased by HtyOle. In addition to that, microRNAs (miRs) involved in both redox status balance and skin regeneration potential were also tested. The following miRs, hsa-miR-21 and hsa-miR-29a, were increased while has-miR-34a was not affected by HtyOle.

## 1. Introduction

**Figure 1:**
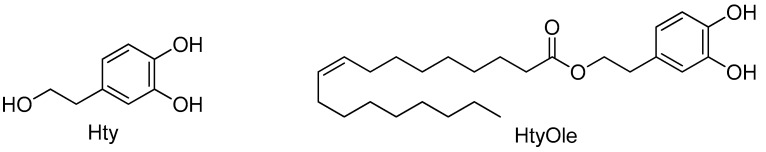
Structure of hydroxytyrosol (Hty) and hydroxytyrosyl oleate (HtyOle)**.**

Hydroxytyrosol (Hty, Figure 1) is one of the main phenolic compounds in olive fruit. It can occur in its free form, or as a moiety in more complex structures, such as secoiridoids (oleuropein and its aglycone). The distribution among the different forms depends on many factors, such as cultivar, ripening stage, and processing and storage conditions, affecting the hydrolytic activity of endogenous enzymes that release Hty from the secoiridoids [1,2]. As in the case of other polar phenolics, Hty is more abundant in the by-products of olive oil production, namely olive mill waste water (OMWW) and pomace, rather than in olive oil itself [3]. Hydroxytyrosol has been reported to display a number of biological activities, including anticancer, antioxidant, and anti-inflammatory properties [2,4,5]. Despite its potential health benefits, its uses in food and cosmetic industries are limited by its hydrophilic character that eventually leads to low bioavailability. Lipophilization has been suggested as a promising strategy to improve the properties of Hty as well as of other polar phenolics [6]. A number of lipophilic derivatives, including esters bearing fatty acyl chains, have been investigated. Indeed, some hydroxytyrosyl esters were found to display enhanced antioxidant activities, compared to parent Hty, and the activity was found to depend on the chain length [7,8,9,10,11,12,13,14,15,16,17,18,19,20,21,22]. The lipophilic character of hydroxytyrosyl esters suggests their potential use as active compounds in epidermal and dermal formulations for the treatment of the inflammation of the cutaneous stratus [23]. Remarkably, we recently reported on the in vitro anti-inflammatory properties of hydroxytyrosyl oleate (HtyOle, Figure 1) [24]. 

Although no correlation has been established yet, several antimicrobial agents have antioxidant capabilities [25]. In particular, hydroxytyrosyl esters show both antioxidant and antimicrobial activities at significant levels [26,27]. Reactive Oxygen Species (ROS) are crucial biochemical mediators for senescence and age-related diseases, including disorders of human skin [28,29]. MicroRNAs (miRs) have been well recognized as biomarkers of pathology (especially in cancer) and regulators of gene expression by nutrients and nutrition special regimen [30,31]. Their role in the regulation of antioxidant defense and oxidative stress is emerging [32,33]. Indeed, dysfunction of the antioxidant defense system and the imbalance between formation and removal of ROS can lead to cell damage due to free radical reactions. Herein we studied the effect of HtyOle as a free radical scavenger on the HaCat human keratinocyte cell line, on ROS formation and antioxidant enzymes, as well as its capability to modulate miRs linked to redox cellular state and skin regeneration.

## 2. Materials and Methods 

### 2.1. Chemicals and Reagents

Hydroxytyrosol was obtained by reducing 3,4-dihydroxyphenylacetic acid with LiAlH_4_ (Sigma-Aldrich, Milan, Italy), as already reported [34]. *n*-Hexane and acetone (analytical grade) were supplied from Carlo Erba Reagenti (Milan, Italy). Methyl oleate and LC/MS grade solvents (methanol and formic acid) were acquired from Sigma-Aldrich (Milan, Italy). Ultrapure water was obtained by Milli-Q plus system (Millipore, Bedford, MA, USA). Novozym®435 (immobilized *Candida antarctica* Lipase B) was from Novozymes (Bagsværd, Denmark). Dulbecco’s modified Eagle’s medium (DMEM), fetal bovine serum (FBS), l-glutamine, penicillin/streptomycin, paraformaldehyde (PFA), and TaqMan microRNA primers were obtained from Thermo Fisher Scientific (Waltham, MA, USA). 

### 2.2. Instrumentation

^1^H NMR and ^13^C NMR analyses were performed at 25 °C on a Bruker AC 300 spectrometer at 300 MHz and 75 MHz, respectively, using chloroform-*d* as the solvent and tetramethylsilane as the internal standard. HPLC analyses were conducted by an Agilent Technologies 1200 series liquid chromatograph provided with a G1379B degasser, a G1312A pump, and a G1329A autosampler. Separations were attained by means of a Discovery C-18 column (3 μm particle size; 150 mm length; 4.6 mm i.d., Merck KGaA, Darmstadt, Germany). MS/MS spectra were recorded by using an API 4000 Q-Trap (AB Sciex, Framingham, MA, USA) mass spectrometer. Detailed instrumental conditions have been previously reported [24].

### 2.3. Chemistry

Hydroxytyrosyl oleate (HtyOle) was synthesized as previously reported [24]. Briefly, Hty was reacted with methyl oleate, with Novozym®435 as the catalyst, in *t*-BuOH. Reaction was conducted in an orbital shaker at 50 °C for 24 h. After filtering off the lipase and evaporating the solvent, column chromatography on silica gel (*n*-hexane-acetone as the eluent) yielded HtyOle. Spectroscopic data were in line with literature [21], and purity was ˃98% as assessed by HPLC. 

### 2.4. Sample Preparation

Pomace and olive mill waste water (OMWW) were obtained by using Oliomio 50 hammer mill (Toscana Enologica Mori, Val di Pesa (FI)) at the Research Center for Olive, Citrus and Fruit Trees (Rende, Italy) starting from 10 kg of olives. Monovarietal extra virgin olive oil (EVOO) was produced from olives of Carolea cultivar in the crop year 2017/2018. All the samples were stored in the refrigerator until analysis.

### 2.5. Extraction of the Phenolic Fraction from Olive Oil and By-Products

Three different methods for the extraction of the phenolic fraction from matrices were used. *Method 1.* The first method was employed in our previous work [24]. In particular, the extraction procedure from olive oil and by-products was carried out according to Bianco and coworkers, using 1/1 MeOH-acetone (*v*/*v*) solution containing 0.5% sodium metabisulfite [35]. *Method 2.* The second method previously reported by Sivakumar and coworkers [36], is a modification of the first one. *Method 3.* The third method for the extraction of phenolic fraction was carried out according to the International Olive Council [37], using 8/2 MeOH-water (*v*/*v*) solution.

### 2.6. Determination of Hydroxytyrosyl Oleate in the Phenolic Fractions Obtained by Different Extraction Methods

The quantitative determination of HtyOle in the phenolic fractions, obtained by means of the three different extraction methods discussed in the previous paragraph, was performed according to our previously published analytical method [24].

### 2.7. Cell Culture

A human immortalized HaCat keratinocyte cell line was obtained from CLS Cell Lines Service GmbH (Germany) after a material transfer agreement and cultured in 75 cm^2^ flasks. Keratinocytes were cultured at 37 °C, 5% CO_2_, in DMEM containing 10% FBS and 1% antibiotics (10,000 μg mL^−1^ streptomycin and 10,000 units∙mL^−1^ penicillin). Cell counting was achieved using a Countess Automated Cell Counter (Thermo Fisher Scientific, Waltham, MA, USA) by Trypan Blue staining. Samples were solubilized in dimethyl sulfoxide (DMSO) and diluted in complete medium in order to reach the final concentration. DMSO concentration in each final treatment was maintained below 0.1% and the untreated control was represented by the same amount of DMSO present in the treatments. For each treatment, cells were plated in a 96-well plate for the MTT assay, 24-well plate for fluorescence microscopy and in 100 mm polystyrene dishes for microRNA analyses (Falcon, Becton-Dickinson, Lincoln Park, NJ, USA). 

### 2.8. Cell Viability Assay

Cell viability was estimated by MTT assay, evaluating the decrease of 3-(4,5-dimethylthiasol-2-yl)-2,4-diphenyltetrazolium bromide (MTT) by mitochondrial succinate dehydrogenase [38]. The absorbance (Abs) was read with a microtiter plate reader (Synergy H1 by BioTeck, Winooski, VT, USA) at 570 nm (test wavelength, *tw*) and at 690 nm (reference wavelength, *rw*). The optical density (OD) was calculated as Abs *_rw_* − Abs *_tw_*.

### 2.9. Fluorescent Staining

Glass cover slips were inserted into 24 well plates and HaCat cells were grown on it. HtyOle pre-treatment at 5 µM with HtyOle for 24 h was performed in order to determine cytosolic ROS formation. After 30 min of UV lamp exposure at 254 nm (UVC spectrum to avoid vitamin D synthesis). CellRox Deep Red (5 µM, λ = ex/em = 640/665) dye was added. Cells were then fixed with PFA (4%) and imaged using the confocal FV-3000 Olympus microscope as previously described [39]. 

### 2.10. Antioxidant Enzymes Activity and Lipid Peroxidation

Cell lysate was sonicated and divided into two equal parts. One aliquot was centrifuged at 10,000 rpm for 5 min and the supernatant was used for glutathione-S-transferase (GST) and superoxide dismutase (SOD) enzymatic activity assays. The second aliquot was used for the lipid peroxidation assay. The enzymatic activity of SOD and GST in the cell extracts was evaluated as previously described [40]. The Lowry method was used to estimate protein concentration in the samples [41]. The extent of lipid peroxidation was evaluated by measuring thiobarbituric acid reactive species (TBARS) formation on acid heating reaction, as previously described [42], with slight modifications. Briefly, the samples were mixed with 5% trichloroacetic acid and 0.7% TBA (1 mL each), then heated at 100 °C for 15 min. The amount of TBARS was evaluated by measuring the absorbance at 535 nm and was expressed as malondialdehyde (MDA) equivalents (nmol mg^−1^ protein^−1^).

### 2.11. MicroRNA Extraction and Loop Primer Method

MicroRNA was isolated using PureLink™ miRNA Isolation Kit (K1570-01 Ambion by Life Technologies) as previously described [30]. The amount of miRNA was quantified with a termed looped primer RT-PCR method. Total RNA (10 ng) was reverse transcribed by polymerase chain reaction using the TaqMan MicroRNA Reverse Transcription kit (Thermo Fisher Scientific, Waltham, MA, USA) for both the miR target and endogenous control, according to manufacturer’s instructions. The thermocycling conditions were: 30 min at 16 °C, followed by 30 min at 42 °C, 5 min at 85 °C and 5 min at 4 °C. 

### 2.12. Quantitative Real Time PCR (qRT-PCR)

The quantitative real-time polymerase chain reaction (qRT-PCR) was achieved by TaqMan Universal PCR Master Mix Kit (Thermo Fisher Scientific, Waltham, MA, USA), according to the manufacturer’s instructions and QuantumStudio3TM Real-Time PCR Systems equipment. The thermocycling conditions used have been previously described [43]. After completion of the qRT-PCR experiments, the average values of the cycle threshold (Ct) of the reactions in triplicate were calculated. The relative expression of the miR target was plotted as follows: 40 total qRT-PCR cycles –Ct target miR. This difference (ΔCt) was plotted directly. 

### 2.13. Statistical Analysis 

Prism GraphPad Prism version 5.0 for Windows (GraphPad Software, San Diego, CA, USA) was used to build graphs. Differences were evaluated by one-way ANOVA, followed by multi-comparison Dunnett’s test (* *p* < 0.05, ** *p* <0.02, *** *p* < 0.01, compared to controls). 

## 3. Results 

### 3.1. Quantification of Hydroxytyrosyl Oleate in Olive Oil and By-Products Subjected to Different Extraction Procedures

Three methods for the extraction of the phenolic fraction from EVOO and olive oil by-products were compared, with respect to the amount of HtyOle. The results are reported in Table 1. Although similar to the first method, the second method of extraction of the phenolic fraction led to higher amounts of HtyOle in both pomace and OMWW. The highest amount of HtyOle was found in the phenolic fraction obtained by the third extraction method in both by-products. Remarkably, by this approach, it was also possible to detect and quantify HtyOle in extra virgin olive oil (EVOO). 

### 3.2. Hydroxytyrosyl Oleate Affects Cell Viability, ROS Formation, SOD and GST Activities and Lipid Peroxidation in Human Keratinocytes

**Figure 2:**
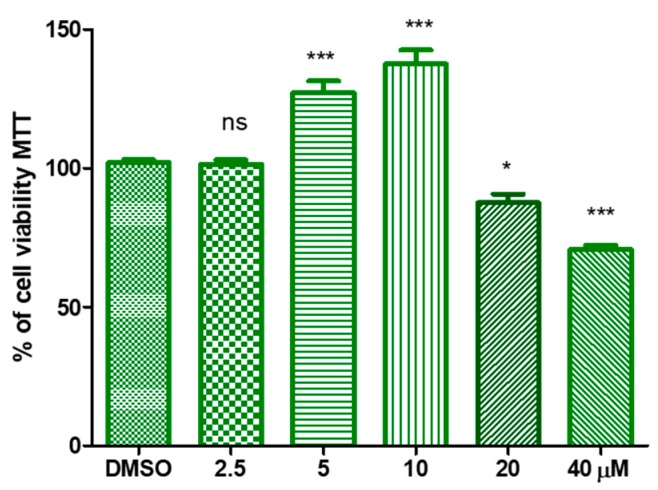
Concentration response of hydroxytyrosyl oleate (HtyOle) in human keratinocyte cell viability. HtyOle significantly affected cell vitality in the range 5–40 µM (* *p* < 0.05; *** *p* < 0.01).

A significant increase in cell vitality was appreciated when HaCat cells were treated with the HtyOle. In particular, a proliferative concentration-dependent action was observed with up to 10 µM of HtyOle, while at 20 and 40 µM cytotoxicity was recorded, as shown in Figure 2. 

**Figure 3:**
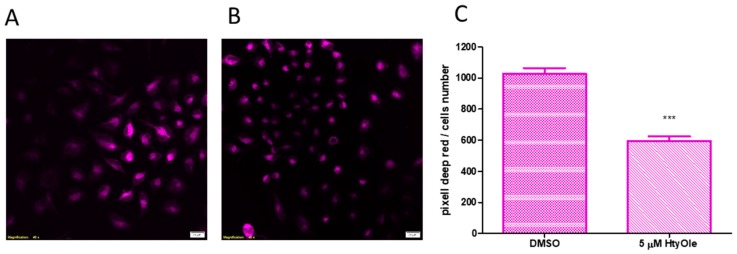
Reactive oxygen species (ROS) formation upon hydroxytyrosyl oleate (HtyOle) treatment in cytosolic compartment. Representative images of ROS stained in keratinocytes cultured without (**A**) and with 5 µM of HtyOle (**B**) for 24 h. Scale bars: 20 µm. Average values of cell area in pixels for red stained keratinocytes (**C**). Data in (**C**) are expressed as the means ± sd of *n* = 3 independent experiments (*** *p* < 0.01).

Therefore, we chose the lower effective concentration of HtyOle (5 µM) for further experiments. To investigate HtyOle’s potential modulation of ROS formation, CellRox Deep Red staining was performed. As shown in Figure 3, keratinocytes cultured for 24 h with HtyOle and then exposed to UV (Figure 3B) showed a decrease of stain intensity with respect to the control (Figure 3A). Figure 3C showed the average values of cell area in pixels for red stained keratinocytes.

**Figure 4:**
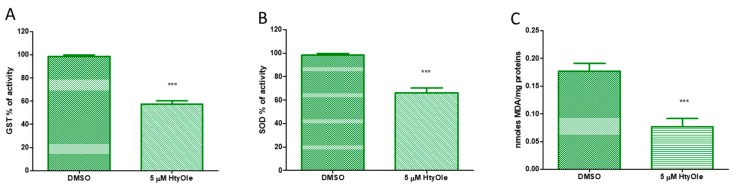
Activity of antioxidant enzymes and lipid peroxidation in human keratinocytes treated with 5 µM of hydroxytyrosyl oleate (HtyOle). Enzymatic activity of Glutathione-S-transferase (GST, (**A**)) and superoxide dismutase (SOD, (**B**)); malondialdehyde (MDA) formation (panel (**C**)). Tests were performed in three independent experiments each done in duplicate (*** *p* < 0.01 versus control).

The potential of HtyOle in modulating the antioxidant defenses in human keratinocytes was also investigated. In our experiments, glutathione-S-transferase (GST) and superoxide dismutase (SOD) activities were decreased by HtyOle (Figure 4, panels A and B, respectively). The oxidative degradation of lipids leads to cell damage. Reactive aldehydes, including malondialdehyde (MDA), are the end products of lipid peroxidation [44]. In our experimental set-up, MDA levels decreased upon treatment with 5 µM of HtyOle when compared to control samples (Figure 4C).

### 3.3. Hydroxytyrosyl Oleate Modulates hsa-miRs Linked to Redox State Status and Human Keratinocyte Regeneration

**Figure 5:**
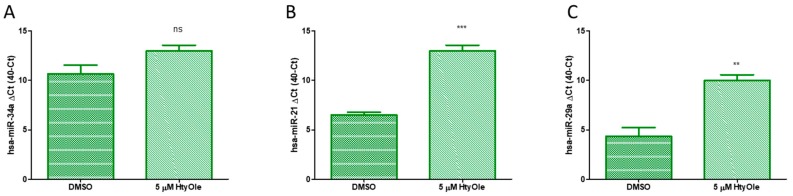
Hydroxytyrosyl (HtyOle) modulates hsa-miR expression linked to redox state and keratinocyte’s regenerative biochemical pathway. Expression of hsa-miR-34a (**A**); hsa-miR-21 (**B**) and hsa-miR-29a (**C**) in the presence or absence of HtyOle. Keratinocytes were cultured with and without HtyOle for 24 h. Tests were performed in three independent experiments each done in duplicate (** *p* < 0.02, *** *p* < 0.01 versus control).

The action of HtyOle on miRs was also investigated to verify whether it can have a skin regeneration potential. Here we tested the following miRs: hsa-miR-34a, hsa-miR-21 and hsa-miR-29a. As shown in Figure 5, up-regulation upon HtyOle treatment was found for hsa-miR-21 (Figure 5B) and hsa-miR-29a (Figure 5C). No significant modulation was recorded for hsa-miR-34a (Figure 5A).

## 4. Discussion

We recently reported the presence of HtyOle in pomace and olive mill waste water (OMWW) [24]. The extraction procedure utilized to recover this hydroxytyrosyl ester from olive oil by-products was the one reported by Bianco and coworkers that allowed the isolation and identification of the analogue tyrosyl oleate (TyOle) from olive drupes of Cassanese cultivar [35]. Herein we show that using a simpler and more economical extraction procedure leads to a higher quantity of HtyOle, both in pomace and in OMWW. Moreover, by means of this approach, it was also possible to identify and quantify HtyOle in EVOO and the results obtained are in line with literature data [45]. This compound was found to be the most active among a series of Hty fatty esters in decreasing nitric oxide (NO) production by lipopolysaccharide (LPS)-stimulated murine macrophages. Moreover, HtyOle suppressed prostaglandin E_2_ (PGE_2_) production and the expression of inducible NO synthase, cyclooxygenase-2 (COX-2) and interleukin-1β (IL-1β) at a transcriptional level [24]. Even though there is not any correlation yet, several anti-inflammatory and antimicrobial agents have antioxidant capabilities [25]. ROS balance is fundamental in all organs and tissues, including skin. In this concern, increased levels of keratinocyte oxidative stress and decreased antioxidant defenses have been correlated with skin-dermal dysfunction [29,46]. Antioxidant defenses of human keratinocytes depend on SOD and GST activities. Both enzymes are involved in the detoxification of the cells from ROS [47]. SOD catalyzes the dismutation of superoxide into oxygen and hydrogen peroxide, and alterations in its activity can lead to unbalanced free radical production. SOD is mainly linked to the mitochondrial redox state [48], while GST is essential to maintain the homeostasis of ROS production and clearance [49]. Our experiments showed that SOD and GST activities are decreased by HtyOle treatment, pointing out its action as free radical scavenger per se. During oxidative stress the cells use their protection apparatus to minimize the process of lipid peroxidation by using the antioxidant enzymes [50]. Therefore, the enzyme activity is shut down because free radicals and lipid peroxidation are dropped [51]. In fact, when cellular redox state is lower also, detoxifications mediated by enzyme activity are resultantly lower [52]. This assumes an important meaning also from a nutritional point of view in skin healing care [53]. The oxidative degradation of lipids results in cell damage. The end products of lipid peroxidation are reactive aldehydes, such as malondialdehyde [44]. In our experimental model, the MDA levels also decreased upon HtyOle treatment when compared to control samples. Recently, the role of nutrition in the regulation of microRNAs (miRs) as well as their capability to regulate cellular redox status and keratinocytes’ regeneration has emerged [31,54]. In fact, it was established that Dicer activity is important for the maintenance of the redox status balance [55]. Moreover, Dicer activation declines with age and Dicer knockout mice might have an impaired antioxidant defense system. We found that hsa-miR-21 and hsa-miR-29a were increased upon HtyOle while hsa-miR-34a was not affected. The physiological action of those miRs is less studied compared to what we know about them in pathophysiology, especially cancer. Of note is that has-miR-29a and hsa-miR-21 are able to regulate skin healing through multiple aspects of this regenerative process. Particularly hsa-miR-21 promoting keratinocytes cell migration [56]. Although increasing levels of this latter miR seem worst in the pathophysiology of many types of cancer and it is considered an oncomiR [57], physiologically, in the context of skin injury and regeneration, it assumes a different and fundamental role. By contrast, hsa-miR34a inhibits proliferation and stimulates apoptosis in human keratinocyte, and it was proposed as a potential therapeutic target for psoriasis [58]. In addition, this miR is suppressed in skin and oral squamous cell carcinomas and in keratinocytes with a compromised differentiation program [59].

## 5. Conclusions

The identification of hydroxytyrosyl oleate in extra virgin olive oil opens a new scenario for the rational use of olive oil in topical formulations. The capability of hydroxytyrosyl oleate to control cellular redox status per se as well as microRNA expression linked to skin regenerative processes, outlines its role as an epigenetic regulator in a plethora of skin related diseases, from healing to aging. 

## Figures and Tables

**Table 1:** Amount of hydroxytyrosyl oleate (HtyOle) depending on extraction procedure.

	Amount of HtyOle (mg kg^−1^)		
	Pomace	OMWW ^1^	EVOO ^2^
Method 1	4.3 ± 0.3 ^a^	2.6 ± 0.2 ^a^	< LOQ ^a^
Method 2	9.3 ± 0.3 ^b^	3.4 ± 0.1 ^b^	< LOQ ^a^
Method 3	16.0 ± 0.2 ^c^	7.1 ± 0.1 ^c^	4.9 ± 0.3 ^b^

^1^ Olive mill waste water (OMWW). ^2^ Extra virgin olive oil (EVOO). Data are expressed as means ± S.D. (*n*= 3). ^a–c^ Different lower case letter superscripts in the same column indicate a significant difference (*p* < 0.05).
